# Knowledge, Attitudes, and Dietary Practices Regarding Low Glycemic Index Foods Among Urban Working Women in Western Province, Sri Lanka

**DOI:** 10.7759/cureus.104797

**Published:** 2026-03-06

**Authors:** Thamashi Jayasinghe, Geeshani Somaratne, Ayesha Nikalansooriya, Umani Walallawita, Dimuthu Gunasekara

**Affiliations:** 1 Nutrition, Postgraduate Institute of Agriculture, University of Peradeniya, Peradeniya, LKA; 2 Food Science and Technology, Faculty of Agriculture, University of Peradeniya, Peradeniya, LKA; 3 Nutrition, Agricultural, Food and Nutritional Science, University of Alberta, Edmonton, CAN; 4 Research Operations, Massey University, Palmerston North, NZL; 5 Research and Development, CIC Dairies, Dambulla, LKA

**Keywords:** blood glucose control, carbohydrate quality, dietary practices, glycemic index, nutrition education, urban women

## Abstract

Background and objective

Carbohydrate quality, particularly glycemic index (GI), plays a critical role in the prevention of non-communicable diseases (NCDs). Despite growing awareness of low-GI diets, evidence on how knowledge and attitudes translate into dietary practices among urban working women in South Asia remains limited. This study assessed knowledge, attitudes, and dietary practices (KAP) related to low-GI foods among urban working women in Western Province, Sri Lanka, and examined associations between KAP domains and sociodemographic characteristics, as well as perceived barriers and facilitators influencing low-GI food consumption.

Methods

A validated, self-administered online questionnaire was used to collect data from 700 urban working women. The tool assessed sociodemographic characteristics and KAP related to low-GI foods and included open-ended questions to explore contextual barriers and facilitators. Knowledge, attitude, and practice scores were categorized as poor, average, or good. Associations were examined using chi-square tests, and qualitative responses were thematically analyzed.

Results

Among participants, 48.1% (337) correctly identified the concept of GI, while only 41.1% (288) recognized the correct low-GI range (<55). Brown rice was identified as a low-GI food by 62.1% (435), whereas only 12.4% (87) correctly recognized white rice as high-GI. Positive attitudes toward low-GI foods were high, with 87.4% (612) agreeing that incorporation promotes health and 86.1% (603) perceiving blood sugar control as important. Interest in learning about low-GI benefits was reported by 94.5% (662), and 81.5% (571) were willing to include low-GI foods in daily meals. Despite this, dietary practices were suboptimal: 45.9% (321) consumed low-GI foods once a week, 32.0% (224) rarely or never, and only 4.7% (33) daily; 58.0% (406) never checked GI values, and 49.4% (346) never read food labels. Associations were observed between knowledge and attitudes (χ² = 43.6, p < 0.05), knowledge and practices (χ² = 68.9, p < 0.05), and attitudes and practices (χ² = 4.0, p < 0.05), highlighting a persistent knowledge-practice gap. Age, education, and income were significantly associated with KAP domains (p < 0.05). Thematic analysis revealed key barriers, including time constraints (work/home responsibilities), limited availability and higher cost of low-GI foods, habitual dietary patterns, taste preferences, and frequent eating outside the home. Facilitators included health awareness, disease prevention motivation, and access to simple nutrition information, practical cooking skills, and availability of convenient low-GI snacks.

Conclusions

Despite adequate knowledge and positive attitudes, translation into low-GI dietary practices remains limited among urban working women. Interventions must move beyond awareness-building to address structural, behavioral, and environmental barriers to improve carbohydrate quality in urban diets.

## Introduction

Non-communicable diseases (NCDs), including type 2 diabetes mellitus, cardiovascular disease, and obesity, represent one of the most significant public health challenges in Sri Lanka, particularly within rapidly urbanizing populations [1,2]. Urbanization, lifestyle transitions, and dietary shifts toward energy-dense and refined carbohydrate-based foods have contributed substantially to the escalating burden of NCDs. Recent national nutrition assessments further highlight a concerning double burden of malnutrition among Sri Lankan women aged 15 to 49 years, with more than half (57.3%) experiencing either undernutrition or overweight/obesity, despite regional and subgroup variations in diet quality [3]. Although some indicators of dietary diversity appear relatively favorable at the national level, the persistent high risk of NCDs suggests that diet quality, particularly carbohydrate quality, remains suboptimal, with a continued preference for high glycemic load foods [4,5].

Within this context, the glycemic index (GI) has gained increasing attention as a useful indicator of carbohydrate quality and a practical dietary tool for the prevention and management of metabolic syndrome and its associated components, including insulin resistance, impaired glucose tolerance, dyslipidemia, hypertension, and central obesity [6]. Low GI foods produce slower and more sustained postprandial increases in blood glucose and insulin concentrations, thereby reducing glycemic variability and metabolic stress. Substantial evidence demonstrates that habitual consumption of low-GI diets improves glycemic control, lipid profiles, and cardiometabolic risk markers, including triglycerides and blood pressure, ultimately reducing the risk of type 2 diabetes mellitus and cardiovascular disease [7-9].

The relevance of low GI dietary strategies is particularly pronounced in Sri Lanka, where rice is the dominant staple food and contributes a major proportion of daily carbohydrate intake. Importantly, the GI of rice-based meals varies considerably depending on rice variety, processing methods, cooking practices, and the composition of accompanying foods [10,11]. Research has shown that traditional Sri Lankan mixed meals, such as boiled red rice consumed with lentil curry, vegetables, and leafy greens, can yield low to moderate GI values, especially when fibre-rich components are incorporated [12]. Furthermore, increasing the dietary fibre content of rice-based meals through the inclusion of locally available fibre sources has been shown to significantly lower their GI, offering a culturally appropriate and sustainable approach to improving glycemic responses without necessitating drastic dietary modifications [10]. Emphasizing low GI food choices within traditional dietary patterns may therefore play a critical role in mitigating the growing burden of metabolic syndrome, particularly among urban populations.

Understanding dietary behavior through the lens of GI is especially important for urban working women, a group that experiences unique nutritional challenges due to time constraints, occupational demands, sedentary lifestyles, and increased exposure to processed and convenience foods. Sri Lanka currently reports one of the highest diabetes prevalence rates in Asia, exceeding earlier estimates of 8% to 15% and surpassing current global averages for Asian countries [2]. While dietary intake patterns are central to the development of diet-related chronic diseases, behavioral determinants such as knowledge, attitudes, and dietary practices (KAP) play a crucial mediating role. Adequate nutrition knowledge supports informed food choices; positive attitudes foster motivation for dietary change; and dietary practices reflect the practical implementation of knowledge and beliefs [13,14]. However, these dimensions do not always align in urban food environments where high-GI, energy-dense foods are widely available, affordable, and socially acceptable.

National dietary surveys and regional studies further illustrate the nutritional vulnerabilities faced by working women in Sri Lanka. Evidence from the Western Province indicates that more than one-third of working women are overweight (38%) or obese (13%), while only a small proportion meet minimum dietary diversity requirements. These patterns are characterized by frequent consumption of sugar-sweetened beverages, tea or coffee with added sugar, and energy-dense snacks [15]. Although awareness of GI was not directly assessed in these studies, the findings underscore broader dietary trends that may contribute to elevated glycemic exposure and increased metabolic risk.

Supporting evidence from other Sri Lankan populations demonstrates significant associations between nutrition-related knowledge, dietary practices, and health outcomes. Among reproductive-age women in marginalized settings, nutrition knowledge and practices have been linked to body mass index and household food security, highlighting the influence of both cognitive and environmental determinants on dietary behavior [16]. Additionally, lifestyle pattern analyses among urban women reveal interactions between dietary behaviors and physical activity levels that influence cardiometabolic risk factors, including waist circumference and blood glucose indices [17,18]. These findings collectively reinforce the interconnected nature of KAP in shaping metabolic health outcomes.

Given the high prevalence of metabolic risk, the dominance of carbohydrate-rich diets, and the rapidly evolving urban food environment, there is a clear need to examine KAP related specifically to low GI foods among urban working women in Sri Lanka. While low-GI dietary patterns are widely recognized for their protective role against type 2 diabetes and cardiovascular disease, their successful adoption depends largely on individual awareness, perceptions, and habitual practices [19]. The aim of this study is to assess the current levels of KAP regarding low-GI foods among urban working women in Sri Lanka and to identify gaps that may hinder the adoption of low-GI dietary habits. The Western Province was selected for this study, as it is the most urbanized and economically active region of Sri Lanka, characterized by a high concentration of working women, greater exposure to processed and convenience foods, and a higher burden of lifestyle-related NCDs. Findings from this research are expected to provide critical insights for designing targeted nutrition education, culturally appropriate interventions, and evidence-based policy strategies aimed at improving diet quality and reducing NCD risk in this important and growing segment of the Sri Lankan population.

## Materials and methods

Study design

This study was conducted as a descriptive cross-sectional survey to assess the KAP related to low GI foods among urban working women in the Western Province of Sri Lanka. A quantitative approach was adopted to obtain a snapshot of GI-related dietary behaviors and associated socio-demographic factors within the target population.

Study population and setting

The study population comprised urban working women aged 20 to 59 years residing in selected urban areas in the Western Province of Sri Lanka. Urban areas were defined according to the criteria of the Department of Census and Statistics, Sri Lanka. Working women were defined as those engaged in full-time or part-time paid employment in either the public or private sector at the time of data collection. Women who were pregnant, lactating, or diagnosed with chronic conditions requiring therapeutic diets (other than diet-controlled diabetes) were excluded to minimize potential dietary bias.

Sample size determination and sampling technique

The minimum sample size was calculated using the standard formula for cross-sectional studies: $n=\frac{Z^{2}p\left( 1-p \right)}{d^{2}}$ where n is the required sample size, Z is the standard normal deviate at 95% confidence level (1.96), p is the assumed prevalence of adequate knowledge or practices related to low GI foods (assumed as 50% due to lack of prior national data), and d is the margin of error (5%). After adjusting for an anticipated non-response rate of 10%, the final sample size was determined accordingly. The minimum required sample size was 384 participants. After adjusting for a 10% anticipated non-response rate, the final sample size was determined to be 422 urban working women in the Western province.

Participants were recruited using a stratified random sampling technique, with strata based on urban districts in the Western Province (Colombo, Gampaha, and Kalutara), employment sector (public/private), and educational level. Recruitment channels included organizational email lists, workplace WhatsApp and messenger groups, and targeted social media platforms. This approach ensured broad socio-demographic representation across age, income, profession, and education, thus enhancing the generalizability of the findings to urban working women in Sri Lanka.

Data collection instruments

Data collection was carried out using a validated, self-administered online questionnaire available in English (see Appendix A), Sinhala, and Tamil. The questionnaire was carefully developed in accordance with the study objectives to comprehensively assess KAP related to low GI carbohydrate-based foods among urban working women in Sri Lanka. In addition to the KAP components, the tool captured key sociodemographic characteristics, including age, marital status, educational attainment, employment status, and household income. Furthermore, open-ended questions were included to explore perceived barriers and facilitators to the consumption of low GI foods, enabling the collection of qualitative insights into participants’ real-life experiences, perceptions, and challenges.

The questionnaire comprised three main components: knowledge, attitudes, and practices related to low GI foods. The knowledge section included 10 multiple-choice questions assessing participants’ understanding of the health benefits, dietary sources, and fundamental principles of low GI foods. Each correct response was awarded 1 mark, while incorrect responses received 0, resulting in a total possible knowledge score ranging from 0 to 10. The attitude section consisted of 16 items assessed using a five-point Likert scale (strongly agree/very confident = 5 to strongly disagree/not confident at all = 1), including 10 main questions with six sub-items. This section evaluated participants’ beliefs, perceptions, and confidence regarding the adoption of low GI dietary practices, with a maximum attainable score of 80. The practice section comprised 12 items (10 main questions with six sub-items under one question) measured on a four-point Likert scale ranging from rarely or never (1) to daily (4). This section assessed dietary behaviors, frequency of low GI food consumption, and related lifestyle practices, yielding a maximum score of 48. The overall KAP score was converted into a percentage, and KAP levels were categorized as good/high (>75%), average (50% to 74%), and poor (<50%).

The reliability and validity of the KAP questionnaire

To ensure the validity and reliability of the questionnaire, a rigorous content validation process was undertaken. The initial drafts were reviewed by a panel of experts in human nutrition, public health, and behavioral sciences (n = 10), who evaluated each item for relevance, clarity, and alignment with the research objectives. Based on their feedback, necessary revisions were made to enhance content accuracy, linguistic clarity, and cultural appropriateness. Subsequently, a pilot study was conducted with 25 (n=25) urban working women who were not part of the main study sample. This pilot phase assessed the clarity, logical flow, and time required to complete the questionnaire and identified any technical issues with the online format. Feedback from the pilot study was used to further refine the questionnaire prior to implementation in the full-scale study.

Ethical considerations

Ethical approval was obtained from the Allied Health Sciences Ethics Review Committee of the University of Peradeniya (approval no. AHS/ERC/2024/069) before the commencement of the study. Informed consent was obtained from all participants, and their anonymity and confidentiality were strictly maintained throughout the study. Participants were assured that their participation was entirely voluntary and that they had the right to withdraw from the study at any stage without facing any consequences.

Statistical analysis

The collected data were exported and analyzed using SPSS Statistics version 21.0 (IBM Corp., Armonk, NY, USA). Descriptive statistics were used to summarize the data, with categorical variables presented as frequencies and percentages, and continuous variables expressed as mean ± standard deviation (SD). Chi-square (χ²) tests were performed to examine the relationships between KAP and selected socio-demographic variables. Furthermore, a Chi-square test was used to assess the association between knowledge levels, attitude levels, and dietary practice levels. All statistical analyses were conducted at a 5% level of significance, and p-values ≤ 0.05 were considered statistically significant.

## Results

Sociodemographic characteristics of the study population

A total of 758 urban, educated working women responded to the initial online survey. After applying predefined eligibility criteria, including age (18 to 60 years), urban residency, education (minimum diploma or undergraduate qualification), professional or semi-professional employment, and exclusion of those with diagnosed diabetes, pregnancy/lactation, or medically prescribed low-carbohydrate diets, a final sample of 700 eligible participants was retained for analysis. The sociodemographic characteristics of the respondents are summarized in Table 1.

**Table 1:** Sociodemographic characteristics of the study population (total n = 700) Data are expressed as frequency and percentage; LKR: Sri Lankan rupees

Characteristic	Category	Frequency	Percentage
Age (years)	18-30	342	48.9
	31-40	135	19.3
	41-50	88	12.6
	51-60	95	13.6
	61-70	40	5.7
Education level	Diploma/Higher National Diploma	84	12.0
	Undergraduate/Graduate	422	60.3
	Postgraduate/Professional	194	27.7
Income (LKR)	< 50,000	240	34.3
	50,000-100,000	231	33.0
	100,000-150,000	171	24.4
	> 150,000	58	8.3
Ethnicity	Sinhala	612	87.4
	Tamil	46	6.6
	Muslim	29	4.1
	Burgher	8	1.1
Marital status	Not married	233	33.3
	Married	441	63.0
	Divorced	13	1.9
	Widowed	13	1.9

Nearly half of the participants were aged between 18 and 30 years (342, 48.9%), representing the largest age group, followed by those aged 31 to 40 years (135, 19.3%). Women aged 51 to 60 years accounted for 13.6% (95) of the sample, while smaller proportions were observed in the 41- to 50-year (88, 12.6%) and 61- to 70-year (40, 5.7%) age categories. With regard to educational attainment, the majority of respondents had completed undergraduate or graduate-level education (422, 60.3%), while 27.7% (194) possessed postgraduate or professional qualifications. A smaller proportion (84, 12.0%) reported holding a diploma or higher national diploma.

In terms of their income, approximately one-third of participants reported a monthly income below Sri Lankan rupee (LKR) 50,000 (240, 34.3%), closely followed by those earning between LKR 50,000 and 100,000 (231, 33.0%). About one-quarter of the sample (171, 24.4%) reported income ranging from LKR 100,000 to 150,000, whereas only 8.3% (58) earned more than LKR 150,000. The ethnic composition was predominantly Sinhala (612, 87.4%), with Tamil (46, 6.6%), Muslim (29, 4.1%), and Burgher (8, 1.1%) participants comprising smaller proportions. Regarding marital status, most respondents were married (441, 63.0%), while one-third were not married (233, 33.3%). Divorced and widowed women each accounted for 1.9% (13) of the study population.

Distribution of KAP

Table 2 summarizes participants’ knowledge of low-GI foods. Less than half of the respondents (337, 48.1%) correctly identified GI as a measure of how quickly foods raise blood glucose levels; notable proportions held misconceptions, including associating GI with protein content (165, 23.6%) or digestion time (130, 18.6%). Knowledge of the correct low-GI range was suboptimal, with only 41.1% (288) correctly identifying foods with a GI value below 55. Regarding characteristics of low-GI foods, 35.6% (249) correctly recognized that they are digested slowly, whereas 31.7% (222) incorrectly believed they contain easily digested sugars.

**Table 2:** Distribution of participants’ knowledge regarding low GI foods (total n = 700) Data are expressed as frequency and percentage; GI: Glycemic index

Variable	Category	Frequency	Percentage
Concept of GI of foods	The GI measures how fast food raises blood sugar	337	48.1
	The GI measures food's protein content	165	23.6
	The GI measures food digestion time	130	18.6
	The GI measures a food's vitamins and minerals	68	9.7
Correct level of low-GI range	GI less than 55	288	41.1
	GI between 56 and 69	176	25.1
	GI above 55	96	13.7
	GI between 40 and 80	140	20.0
Characteristic not in low-GI food	High fiber diet has a low GI	172	24.6
	Low GI foods digest slowly	249	35.6
	Low GI foods have easily digested sugars	222	31.7
	Low GI foods aid fullness, lower diabetes risk	57	8.1
Which food has a high GI?	White rice	87	12.4
	Green vegetables	165	23.6
	Sausages	351	50.1
	Oatmeal	97	13.9
How does GI help diabetes?	By choosing foods that cause a quick rise in blood sugar levels	57	8.1
	By selecting foods that stabilize blood sugar levels	485	69.3
	By avoiding all carbohydrates	100	14.3
	By increasing overall calorie intake	58	8.3
Which food has low GI?	White bread	45	6.4
	Brown rice	435	62.1
	French fries	78	11.1
	Watermelon	142	20.3
Which hormone controls blood sugar?	Insulin	508	72.6
	Adrenalin	66	9.4
	‍Testosterone	75	10.7
	Growth hormone	51	7.3
Which traffic light color indicates low sugar?	Yellow	293	41.9
	Green	318	45.4
	Red	41	5.9
	Orange	48	6.9
Which of the following is an incorrect statement?	Parboiled rice contains a higher GI than raw rice	242	34.6
	Parboiled rice contains a lower GI than raw rice	321	45.9
	Steaming lowers the GI of sweetpotato	94	13.4
	Frying/making fried rice increases the GI of rice ‍	43	6.1
Which of the following foods are bad for diabetics?	Brown rice and Basmati rice	21	3.0
	Pasta and yoghurt	122	17.4
	Polished white rice and sugary foods	544	77.7
	Soy products and lentils	13	1.9

Half of the participants (351, 50.1%) incorrectly identified sausages as a high-GI food, whereas only 12.4% (87) correctly identified white rice as the high-GI option. In contrast, a majority of respondents (485, 69.3%) correctly understood that the GI is useful in diabetes management by helping to stabilize blood glucose levels. Brown rice was correctly identified as a low-GI food by 62.1% (435) of respondents. Most participants (508, 72.6%) correctly identified insulin as the hormone regulating blood glucose. However, mixed understanding was observed regarding cooking methods and GI, and only 45.9% (321) correctly identified the incorrect statement related to parboiled rice. Overall, the findings indicate moderate knowledge with persistent gaps and misconceptions regarding low-GI foods.

Overall, respondents demonstrated highly positive attitudes towards low GI foods (Table 3). A substantial majority agreed or strongly agreed that incorporating low GI foods promotes health (612, 87.4%) and aids weight loss and overall well-being (605, 86.4%). Blood sugar control was perceived as very or somewhat important by 86.1% (603) of participants. Interest in learning about low GI benefits was notably high, with 94.5% (662) expressing interest and 81.5% (571) reporting willingness or openness to include low GI foods in daily meals. However, confidence in identifying low GI foods was moderate, as over half of the respondents (375, 53.6%) reported a neutral level of confidence. Cost and taste emerged as key perceived barriers; 67.7% (474) believed low-GI foods were costlier, and 47.0% (329) perceived them as less flavorful compared to high-GI foods. Regarding reasons for consumption, strong agreement was observed for chronic disease prevention, diabetes management, weight management, and control of blood sugar, blood pressure, and cholesterol, indicating that health-related motivations strongly underpin positive attitudes towards low GI foods.

**Table 3:** Distribution of participants’ attitudes regarding low GI foods (total n = 700) Data are expressed as frequency and percentage; GI: Glycemic index

Variable	Category	Frequency	Percentage
Incorporating low-GI foods promotes health	Strongly agree	420	60.0
	Agree	192	27.4
	Neutral	66	9.4
	‍Disagree	17	2.4
	Strongly disagree	5	0.7
Confident level of identifying low-GI foods	Very confident	45	6.4
	Confident	164	23.4
	Neutral ‍	375	53.6
	Not confident	98	14.0
	Not confident at all	18	2.6
Low-GI foods aid weight loss and health	Strongly agree	393	56.1
	Agree	212	30.3
	Neutral ‍	87	12.4
	Disagree	6	0.90
	Strongly disagree	2	0.30
Low-Gi foods are important for the level of blood sugar control	Very important	329	47.0
	Somewhat important	274	39.1
	Neutral ‍	69	9.9
	Normally not important	27	3.9
	Not important at all	1	0.1
Interested in learning low-GI benefits	Very interested	549	78.4
	Somewhat interested	113	16.1
	Neutral ‍	36	5.1
	Not interested	2	0.3
Willing to add low-GI foods to daily meals	Definitely (already prioritized) ‍	87	12.4
	I'm open to trying low-GI foods	484	69.1
	Maybe. if it's convenient	106	15.1
	No, I prefer my current diet	15	2.1
	I haven’t considered it /Not at all	8	1.1
Low-GI foods costlier than high-GI food	Yes, definitely	284	40.6
	Yes, but it's worth the cost	190	27.1
	No, prices are similar	177	25.3
	No, they're cheaper	43	6.1
	No, they're very cheap	6	0.9
How convenient is preparing low-GI meals?	Very convenient	140	20.0
	Somewhat convenient	276	39.4
	Neutral ‍	256	36.6
	Inconvenient	22	3.1
	Very inconvenient	6	0.9
Taste of low-GI food compared to high-GI foods	More flavorful	23	3.3
	Flavorful	162	23.1
	About the same	186	26.6
	Less flavorful	311	44.4
	Very little flavor	18	2.6
To reduce non-communicable disease risk	Strongly agree	399	57.0
	Agree	210	30.0
	Neutral ‍	90	12.9
	Disagree	0	0.0
	Strongly disagree	1	0.1
To avoid present health issues	Strongly agree	153	21.9
	Agree	410	58.6
	Neutral ‍	123	17.6
	Disagree	7	1.0
	Strongly disagree	7	1.0
High nutritional values and health benefits	Strongly agree	372	53.1
	Agree	207	29.6
	Neutral ‍	117	16.7
	Disagree	3	0.4
	Strongly disagree	1	0.1
To maintain better health	Strongly agree	417	59.6
	Agree	195	27.9
	Neutral ‍	87	12.4
	Disagree	0	0.0
	Strongly disagree	1	0.1
To control blood sugar, pressure, and cholesterol	Strongly agree	425	60.7
	Agree	226	32.3
	Neutral ‍	46	6.6
	Disagree	1	0.1
	Strongly disagree	2	0.3
For weight management	Strongly agree	411	58.7
	Agree	231	33.0
	Neutral ‍	54	7.7
	Disagree	2	0.3
	Strongly disagree	2	0.3
Diabetes management	Strongly agree	423	60.4
	Agree	220	31.4
	Neutral ‍	49	7.0
	Disagree	5	0.7
	Strongly disagree	3	0.4

As depicted in Table 4, participants’ dietary practices regarding low GI foods were generally suboptimal. Nearly half of the respondents consumed low GI foods only once a week (321, 45.9%), while 32.0% (224) reported rare or no consumption, and only 4.7% (33) consumed them daily. More than half had never checked the GI of foods (406, 58.0%) or read food labels for GI information (346, 49.4%). Meal planning with low GI foods was mainly occasional (323, 46.1%), with very few practicing this daily (23, 3.3%). Although intake of whole grains, legumes, fruits, and vegetables occurred mostly once a week (373, 53.3%), daily consumption remained low (69, 9.9%). Processed foods and sugary snacks were commonly consumed several times per week or occasionally. Despite these patterns, interest in low GI snacks was high, and most participants perceived incorporating low GI foods as challenging but achievable.

**Table 4:** Distribution of participants’ practices regarding low GI foods (total n = 700) Data are expressed as frequency and percentage; GI: Glycemic index

Variable	Category	Frequency	Percentage
How often do you eat low-GI foods?	Rarely or never	224	32.0
	Once a week	321	45.9
	Several times per week	122	17.4
	Daily	33	4.7
Have you searched low-GI foods online?	Never	240	34.3
	Rarely	209	29.9
	Sometimes	205	29.3
	Always	46	6.6
How often do you eat whole grains, legumes, fruits, and vegetables?	Rarely or never	19	2.7
	Once a week	373	53.3
	Several times a week	239	34.1
	Daily	69	9.9
How often do you check the GI of foods?	Never	406	58.0
	Occasionally	214	30.6
	Sometimes	66	9.4
	Always	14	2.0
Do you plan meals with low-GI foods?	Never	143	20.4
	Occasionally	323	46.1
	Sometimes/whenever possible	211	30.1
	Always / Daily	23	3.3
Do you read labels for GI?	Never	346	49.4
	Rarely	222	31.7
	Sometimes	105	15.0
	Always	27	3.9
Consumption frequency of processed foods and sugary snacks	Never	28	4.0
	Occasionally	336	48.0
	Several times per week	282	40.3
	Daily	54	7.7
Do you usually eat out for main meals?	Never	59	8.4
	Occasionally	392	56.0
	Several times per week	210	30.0
	Daily	39	5.6
Do you prefer low-GI snacks like nuts and seeds?	Never	6	0.9
	Somewhat interested	94	13.4
	Interested	252	36.0
	Very interested	348	49.7
Is it easy to add low-GI foods to meals?	Very challenging	41	5.9
	Can be challenging (I'm trying)	378	54.0
	Relatively easy	240	34.3
	Very easy	41	5.9
Consumption frequency of processed meat and high-fat dairy	Rarely or never	46	6.6
	Occasionally	326	46.6
	Several times a week	267	38.1
	Daily	61	8.7
Do you usually snack on fruits or vegetables?	Rarely or never	26	3.7
	Occasionally	169	24.1
	Several times a week	340	48.6
	Daily	165	23.6

Association between KAP and low-GI foods

**Figure 1:**
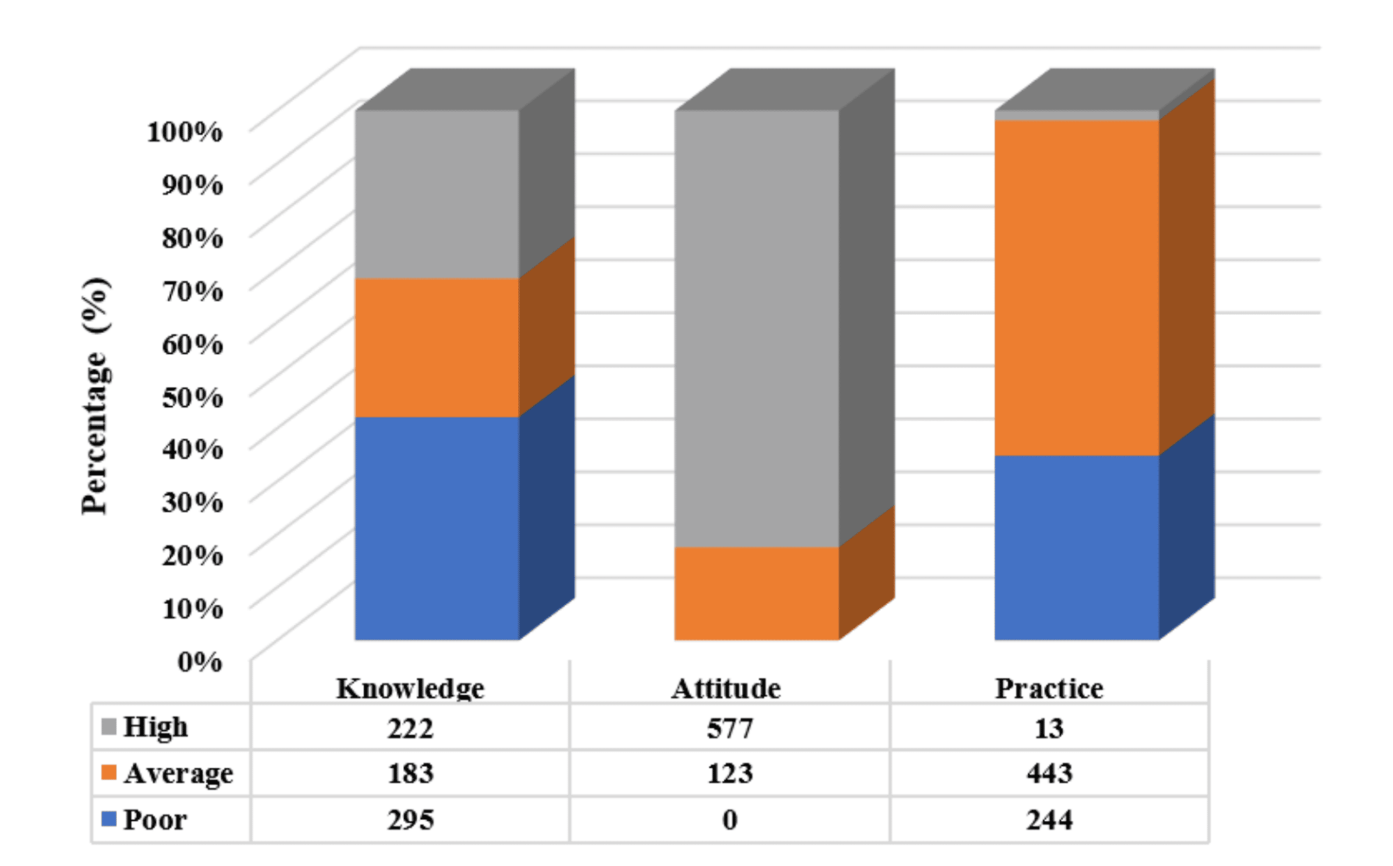
Distribution of knowledge, attitudes, and practices related to low-GI foods GI: Glycemic index; Illustration created by author Thamashi Jayasinghe using Microsoft Excel (Microsoft Corp., Redmond, WA, USA)

The distribution of KAP related to low-GI foods among urban working women is presented in Figure 1. Overall, knowledge regarding low-GI foods was limited among the study population. Less than one-third of respondents (222, 31.7%) demonstrated a high level of knowledge, while 42.1% (295) had poor knowledge and 26.1% (183) exhibited an average level of knowledge. These findings indicate substantial gaps in awareness and understanding of low-GI foods among a large proportion of participants.

In contrast, attitudes toward low-GI foods were overwhelmingly positive. The majority of respondents (577, 82.4%) demonstrated good attitudes, while 17.5% (123) exhibited average attitudes. Notably, none of the participants were classified as having poor attitudes, suggesting a generally favorable perception of low-GI foods across the study population. Despite positive attitudes, dietary practices related to low-GI foods were suboptimal. Only 1.9% (13) of respondents reported good dietary practices, whereas 63.3% (443) demonstrated average practices and 34.9% (244) exhibited poor practices. This marked discrepancy between attitudes and practices highlights a substantial gap between favorable perceptions and actual dietary behavior.

Associations between KAP related to low-GI foods were examined to elucidate the interrelationships among these behavioral domains (Table 5). Knowledge level was significantly associated with attitudes toward low-GI foods (χ² = 43.6, p < 0.05), with higher knowledge corresponding to a greater likelihood of demonstrating favorable attitudes. Although positive attitudes were generally prevalent, the proportion of respondents with good attitudes increased progressively with increasing knowledge, indicating that greater awareness is associated with more favorable perceptions of low-GI foods.

**Table 5:** Association between knowledge, attitudes, and dietary practices df: Degree of freedom

Variable 1	Variable 2	Chi-square (χ²)	df	p-value	Effect size	Observation
Knowledge	Attitude	43.6	2	<0.05	0.25	Higher knowledge → better attitudes
Knowledge	Practice	68.9	4	<0.05	0.22	High knowledge → better practices, but good practice remains low
Attitude	Practice	4.0	6	<0.05	0.08	Good attitudes → more average/good practices, gap persists

Knowledge level was also significantly associated with dietary practices (χ² = 68.9, p < 0.05). Participants with higher knowledge exhibited relatively better practices compared to those with poor knowledge; however, the overall prevalence of good practices remained low across all knowledge categories. A considerable proportion of respondents with high knowledge continued to report average or poor practices, suggesting that knowledge alone is insufficient to drive sustained behavioral change. Attitudes were significantly associated with dietary practices (χ² = 4.0, p < 0.05), with respondents demonstrating good attitudes more likely to report average or good practices. Nevertheless, even among those with favorable attitudes, good practices were uncommon, highlighting a persistent gap between knowledge and attitudes and actual adoption of low-GI dietary behaviors.

Association between sociodemographic characteristics and knowledge levels

As shown in Table 6, knowledge of low-GI foods was significantly associated with age, educational attainment, and monthly family income (p < 0.05). Knowledge levels declined steadily with increasing age, with women aged 18 to 30 years comprising the majority of participants with high knowledge, while poor knowledge was increasingly concentrated among women aged 51 years and above. Educational attainment showed a strong positive gradient: respondents with postgraduate or professional qualifications predominantly exhibited high knowledge, whereas those with diploma or higher national diploma qualifications were overrepresented in the poor knowledge category. A similar socioeconomic gradient was observed for income, with higher knowledge levels concentrated among women from higher-income households and poor knowledge most prevalent among those earning below LKR 50,000 per month. These findings underscore the combined influence of age, education, and economic capacity on knowledge acquisition related to low-GI foods.

**Table 6:** Association between sociodemographic characteristics and knowledge levels df: Degree of freedom

Sociodemographic variable	Knowledge level	Poor (n)	Average (n)	High (n)	χ²	df	p-value	Effect size
Age (years)	18-30	102	63	177	147.7	8	<0.05	0.33
	31-40	73	30	32				
	41-50	50	31	7				
	51-60	45	44	6				
	61-70	25	15	0				
Education	Diploma/Higher National Diploma	50	31	3	194.6	4	<0.05	0.37
	Undergraduate	218	121	83				
	Postgraduate	27	31	136				
Monthly income (LKR)	<50,000	134	90	16	205.9	6	<0.05	0.38
	50,000-100,000	120	55	56				
	100,000-150,000	32	23	116				
	>150,000	9	15	34				

Association between sociodemographic characteristics and attitude levels

Table 7 shows that attitudes toward low-GI foods were significantly associated with age, education, and monthly family income (p < 0.05). Although no respondents demonstrated poor attitudes, marked variation existed between average and good attitude levels. Older women, particularly those aged 51 to 60 years, exhibited the highest prevalence of good attitudes, while younger women aged 18 to 30 years were more likely to demonstrate average attitudes despite higher knowledge levels. Educational attainment was positively associated with favorable attitudes, with postgraduate-qualified respondents showing the strongest positive attitudes. Similarly, the proportion of good attitudes increased progressively with rising income. These findings suggest that positive perceptions of low-GI foods may strengthen with life experience and socioeconomic stability, rather than knowledge alone.

**Table 7:** Association between sociodemographic characteristics and attitude levels df: Degree of freedom; LKR: Sri Lankan rupees

Sociodemographic variable	Attitude level	Average (n)	Good (n)	χ²	df	p-value	Effect size
Age (years)	18-30	61	281	15.5	4	<0.05	0.15
	31-40	36	99				
	41-50	12	76				
	51-60	7	88				
	61-70	7	33				
Education	Diploma/Higher National Diploma	22	62	13.4	2	<0.05	0.14
	Undergraduate	82	340				
	Postgraduate	19	175				
Monthly income (LKR)	<50,000	60	180	24.7	3	<0.05	0.19
	50,000-100,000	44	187				
	100,000-150,000	11	160				
	>150,000	8	50				

Association between sociodemographic characteristics and dietary practice levels

As presented in Table 8, dietary practices related to low-GI foods were significantly associated with age, education, and monthly family income (p < 0.05). Poor practices were most prevalent among younger women aged 18-30 years, while average practices predominated among women aged 31-60 years. Across all age groups, good practices were uncommon, indicating limited translation of knowledge and attitudes into consistent dietary behavior. Educational attainment showed a non-linear association with practice, as respondents with postgraduate or professional qualifications exhibited a comparatively higher prevalence of poor practices, despite high knowledge and favorable attitudes. Income was also significantly associated with practice levels; women in the highest income category demonstrated the greatest proportion of good practices, although overall adherence remained low. Collectively, these findings highlight a persistent knowledge-practice gap, suggesting that structural, behavioral, and environmental barriers may constrain the adoption of low-GI dietary practices, even among socioeconomically advantaged women.

**Table 8:** Association between sociodemographic characteristics and dietary practice levels df: Degree of freedom; LKR: Sri Lankan rupees

Sociodemographic variable	Practice level	Poor (n)	Average (n)		Good (n)	χ²	df	p-value	Effect size
Age (years)	18-30	170		165	7	68.6	8	<0.05	0.22
	31-40	28		105	2				
	41-50	19		68	1				
	51-60	20		72	3				
	61–70	7		33	0				
Education	Diploma/Higher National Diploma	31		52	1	60.6	4	<0.05	0.21
	Undergraduate	105		311	6				
	Postgraduate	108		80	6				
Monthly income (LKR)	<50,000	44		194	2	137.8	6	<0.05	0.31
	50,000-100,000	82		146	3				
	100,000-150,000	112		57	2				
	>150,000	6		46	6				

Qualitative insights

Barriers and Facilitators to Low GI Food Consumption

**Figure 2:**
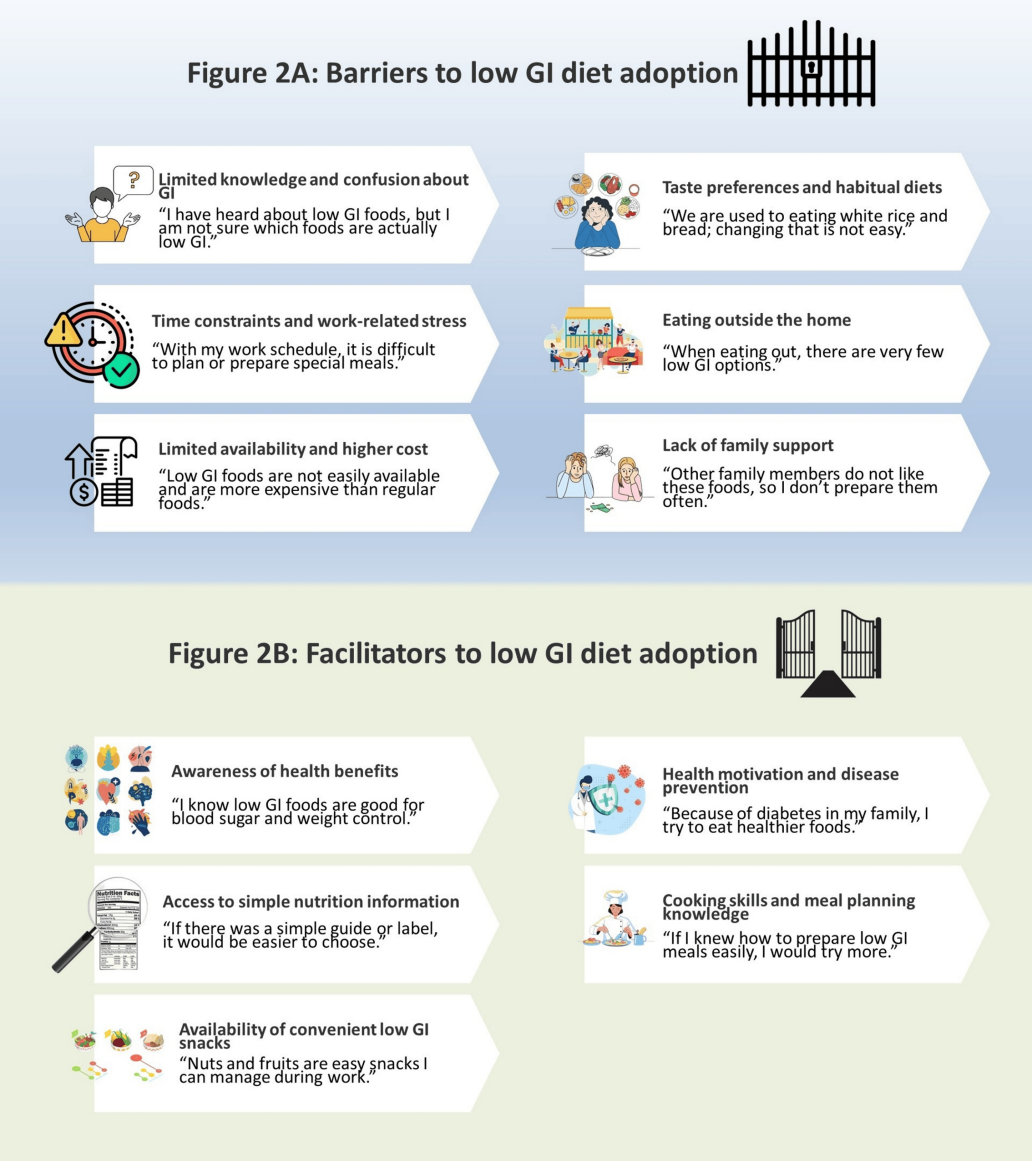
Perceived barriers and facilitators to low-GI food consumption among study participants GI: Glycemic index Illustration created by author Ayesha Nikalansooriya using Microsoft PowerPoint (Microsoft Corp., Redmond, WA, USA) with graphical elements sourced from Freepik (www.freepik.com) and modified by the author.

Thematic analysis of open-ended responses revealed several key factors influencing participants’ consumption of low-GI foods (Figure 2, panel A). Participants commonly reported limited awareness and uncertainty regarding the identification of low-GI foods, including confusion about GI values and food classification. Time constraints related to work schedules and household responsibilities were frequently cited as barriers to meal planning and preparation of low-GI meals. Many respondents highlighted the limited availability and higher cost of low-GI food options, particularly in urban food outlets and workplaces. Taste preferences and habitual dietary patterns, especially reliance on refined carbohydrates and convenience foods, were also noted. Additional barriers included insufficient label information, lack of family support, eating outside the home, and difficulty modifying traditional carbohydrate-based meals.

Facilitators included awareness of health benefits, particularly for weight management, diabetes prevention, and sustained energy levels (Figure 2, panel B). Participants indicated that access to clear nutrition information, such as simple GI guides, food labels, and online resources, would support healthier choices. Availability of affordable low-GI alternatives, workplace or family encouragement, and practical cooking knowledge were also viewed as enabling factors. Many respondents expressed interest in low-GI snacks, such as nuts, seeds, fruits, and legumes, as convenient options. Motivation driven by existing health conditions or preventive health goals further encouraged the adoption of low-GI dietary practices.

Despite relatively high knowledge and generally favorable attitudes toward low-GI foods, qualitative findings reveal multiple structural and behavioral barriers that limit translation into practice. Although participants were aware of the health benefits of low-GI diets, uncertainty in identifying appropriate foods, limited time for meal preparation, and constraints related to cost and availability impeded consistent adoption. Work-related lifestyles and reliance on eating outside the home further reduced opportunities to apply knowledge in daily food choices. Additionally, entrenched taste preferences and lack of family support reinforced habitual consumption of high-GI staple foods. These findings help explain the observed knowledge-practice gap, indicating that awareness alone is insufficient to drive behavior change without supportive food environments, practical skills, and contextual enablers. Addressing these barriers through targeted nutrition education, improved food labeling, and increased accessibility of affordable low-GI options may be essential for improving dietary practices.

## Discussion

To the best of our knowledge, this is the first study in Sri Lanka to provide comprehensive evidence on KAP related to low-GI foods among urban working women. The findings reveal a notable disconnect between positive perceptions and actual dietary behavior, with generally favorable knowledge and attitudes, but consistent adoption of low-GI dietary practices is uncommon. These results are particularly significant in the context of Sri Lanka’s rapidly urbanizing food environment, where refined carbohydrate consumption remains high, and the prevalence of NCDs continues to increase [3,20]. The study underscores the need for interventions that move beyond awareness-raising to address structural, behavioral, and environmental barriers to improve carbohydrate quality and support healthier dietary patterns.

Knowledge related to low-GI foods was significantly associated with age, education, and income, with younger, more educated, and higher-income women demonstrating greater awareness. This aligns with previous evidence indicating that education and socioeconomic status are key determinants of nutrition-related knowledge [16,21]. However, younger women, despite higher knowledge, exhibited poorer dietary practices than older age groups. This discordance highlights the limitations of knowledge-based models of behavior change and underscores the influence of time scarcity, occupational demands, and convenience-oriented food choices among working-age women [15].

Educational attainment showed a strong positive association with knowledge and attitudes, yet its relationship with dietary practices was non-linear. Notably, women with postgraduate or professional qualifications exhibited a relatively high prevalence of poor practices despite demonstrating high knowledge and favorable attitudes. Similar patterns have been reported in other urban populations, where professional employment is associated with irregular meal patterns, frequent eating outside the home, and reliance on ultra-processed foods [22,23]. These findings suggest that occupational context may override individual-level cognitive advantages, limiting the translation of nutrition knowledge into practice.

Household income demonstrated a graded association with knowledge and attitudes, yet higher income did not consistently predict healthier practices. Although women in the highest income category showed a slightly greater prevalence of good practices, overall adherence to low-GI behaviors remained low. This finding reinforces evidence that economic capacity alone is insufficient to ensure healthier dietary choices in urban food systems characterized by widespread availability and social normalization of high-GI, energy-dense foods [16,18]. In Sri Lanka, the central role of polished white rice as a staple further constrains dietary flexibility, even among socioeconomically advantaged groups [4].

The observed associations between KAP were statistically significant but modest in magnitude, reflecting a persistent knowledge-practice gap. While higher knowledge was associated with better attitudes and relatively improved practices, good practices were uncommon across all knowledge and attitude categories. This pattern mirrors findings from studies on diabetes prevention and healthy eating, where positive attitudes fail to translate into sustained behavior change due to environmental and structural barriers [24]. These results challenge linear KAP assumptions and support the need for more comprehensive behavioral frameworks that account for contextual constraints.

Qualitative findings from this study provide critical insight into the mechanisms underlying this gap. Time constraints, work-related stress, and eating outside the home emerged as dominant barriers to low-GI food consumption, consistent with evidence from other urban low- and middle-income country (LMIC) settings undergoing rapid nutrition transition [25]. Limited availability and the perceived higher cost of low-GI foods further restricted adoption, particularly in workplaces and urban food outlets where GI information is rarely available. These findings underscore the role of the food environment in shaping dietary behavior and the inadequacy of individual-level education in isolation.

Taste preferences and habitual reliance on refined carbohydrates, particularly white rice, were also prominent barriers. Cultural food practices strongly influence carbohydrate choices, and modifying staple-based meals is often perceived as difficult or socially undesirable [5,25]. Additionally, lack of family support reduced women’s capacity to sustain dietary changes, reinforcing evidence that household dynamics play a critical role in women’s nutrition behaviors [26].

In contrast, facilitators identified in this study, such as awareness of health benefits, disease-prevention motivation, and interest in convenient, low-GI snack options, represent important intervention entry points. The acceptability of fruits, legumes, nuts, and seeds as snacks suggests that promoting culturally familiar, minimally processed low-GI foods may be more feasible than advocating for major dietary restructuring. Participants’ expressed need for simple, practical nutrition information highlights the importance of actionable guidance, such as clear food labeling, meal-planning tools, and culturally adapted recipes, rather than abstract dietary recommendations [27].

From a public health perspective, these findings have important implications. Interventions targeting urban working women should move beyond awareness-building to address workplace food environments, time constraints, and affordability. Workplace-based nutrition initiatives, improved food labeling policies, and increased availability of affordable low-GI options in urban food outlets may help bridge the knowledge-practice gap. Integrating low-GI principles into national dietary guidelines and public procurement policies could further support healthier carbohydrate choices at the population level. Overall, this study highlights that improving carbohydrate quality among urban working women requires multi-level strategies that align individual knowledge with supportive food environments and social contexts. Such approaches are essential for reducing NCD risk in rapidly urbanizing settings such as Sri Lanka.

Limitations and prospects

This study has a few limitations that should be considered when interpreting the findings. The cross-sectional design precludes causal inferences between KAP. Data were self-reported and collected through an online questionnaire, which may be subject to recall and social desirability bias. The study sample comprised urban, educated, working women, limiting generalizability to rural populations, men, and women with lower educational attainment. Additionally, dietary practices were assessed using frequency-based measures rather than objective dietary assessments.

Future research should employ longitudinal or intervention-based designs to examine causal pathways and behavior change over time. Incorporating objective dietary assessment methods and expanding studies to diverse population groups will strengthen evidence. Importantly, future interventions should adopt multi-level strategies that align individual knowledge with supportive food environments, policy initiatives, and social contexts to improve carbohydrate quality and reduce NCD risk in rapidly urbanizing settings such as Sri Lanka.

## Conclusions

This study demonstrates that urban working women in Sri Lanka possess moderate-to-high knowledge and largely positive attitudes toward low-GI foods; however, the adoption of consistent low-GI dietary practices remains limited. Significant associations between KAP indicate that awareness contributes to favorable perceptions, yet structural, behavioral, and environmental barriers constrain the translation of knowledge into action. Time constraints, workplace food environments, cost and availability of low-GI foods, habitual dietary patterns, and limited family support emerged as key impediments, explaining the observed knowledge-practice gap. These findings suggest that nutritional education alone is insufficient to improve carbohydrate quality in urban diets. Effective interventions should combine targeted education with environmental and policy-level strategies, including improved food labeling, workplace-based nutrition initiatives, and increased access to affordable, culturally appropriate low-GI foods. Addressing these multi-level determinants is critical for promoting sustainable dietary change and reducing the growing burden of NCDs among urban women in Sri Lanka.

## Appendices

Appendix A

Presented below is a copy of the English translation of the validated, self-administered online questionnaire used to collect data for this study.

1. Consent Form

Mark only one oval.

I read and understood the information in the study. I agree to participate in the study.

I read and understood the information in the study. I do not agree to participate in the study.

2. Sociodemographic Characteristics Questionnaire

I. Age

Mark only one oval.

a) Below 18

b) 18 - 30

c) 31 - 40d) 41 - 50

e) 51 -60

f) 61 -70

g) Above 70

II. Marital status*

Mark only one oval.

a) Not married

b) Married

c) Divorced

d) Widowed

e) Any other

III. Ethnicity

Mark only one oval.

a) Sinhala

b) Tamil

c) Muslim

d) Burgher

e) Other

IV. Education Level

Mark only one oval.

a) Non-Education / No formal education

b) Primary Education (1-5 years)

c) Secondary Education (6-11 years)

d) Higher School (College/ 12-13 years)

e) Diploma / HND level education

f) Higher Education - Undergraduate/Graduate level

g) Postgraduate level and/or Professional Qualifications

V. Employment

Mark only one oval.

a) Employed (Government sector)

b) Employed (Private sector)

c) Self-employed

d) Unemployed

e) Student

f) Retired

VI. Monthly Family Income (in local currency - Rs/LKR)

Mark only one oval.

a) Below 50,000

b) 50,000 - 100,000

c) 100,000 - 150,000

d) Above 150,000

VII. Geographical Location:*

Mark only one oval.

a) Rural

b) Semi Urban

c) Urban

3. Dietary Habits and Health

I. Do you currently follow any specific dietary restrictions or special diets?

Mark only one oval.

Yes

No

II. Do you have any of the following non-communicable diseases?

Check all that apply.

- Obesity

- Type-2 Diabetes

- Heart Disease

- Hypertension

- Cholesterol

- Cancer

- No disease condition

III. Do your family members or close relatives have any of the following non-communicable

diseases?

Check all that apply.

- Obesity

- Type-2 Diabetes

- Heart Disease

- Hypertension

- Cholesterol

- Cancer

- No disease condition

4. Knowledge about low glycemic index (GI) Foods

Kindly provide the answers for this section according to your knowledge.

I. What is the concept of the GI of foods?*

Mark only one oval.

a) The GI measures how quickly a food causes blood sugar levels to rise after

consumption.

b) The GI indicates the amount of protein in a food.

c) The GI measures how long it takes for food to be digested.

d) The GI measures the amounts of vitamins and minerals in a food.

II. Which of the following represents the GI range for low-GI foods?*

Mark only one oval.

a) GI less than 55

b) GI between 56 and 69

c) GI above 55

d) GI between 40 and 80

III. Which of the following characteristics is not associated with low-GI food?

Mark only one oval.

a) High-fiber diet has a low GI

b) Low GI foods are digested more slowly and contain more slowly digestible carbohydrates

c) Low GI foods contain highly digestible sugars and cause a fast blood glucose response

d) Low GI foods may help with feeling full and reduce the risk of diabetes

IV. Which of the following foods has a high GI?

Mark only one oval.

a) Oatmeal

b) Green vegetables

c) White rice

d) Sausages

V. How can understanding the GI of foods help with managing diabetes?

Mark only one oval.

a) By choosing foods that cause a quick rise in blood sugar levels

b) By selecting foods that stabilise blood sugar levels

c) By avoiding all carbohydrates

d) By increasing overall calorie intake

VI. Which of the following foods is typically considered low-GI?

Mark only one oval.

a) White bread

b) Brown rice

c) French fries

d) Watermelon

VII. Which of the following hormones is generally considered to control blood sugar?

Mark only one oval.

a) Insulin

b) Adrenalin

c) Testosterone

d) Growth hormone

VIII. Which of the following is the correct traffic light colour to indicate low sugar food per the traffic light colour system for food labelling?

Mark only one oval.

a) Yellow

b) Green

c) Red

d) Orange colour

IX. Which of the following is an incorrect statement?

Mark only one oval.

a) Parboiled rice contains high glycemic index than raw rice

b) Parboiled rice contains low glycemic index than raw rice

c) Steaming significantly lowers the glycemic index of sweet potato

d) Frying/Making fried rice increases the glycemic index of rice

X. Which of the following foods is not good for a diabetes patient?*

Mark only one oval.

a) Brown rice and Basmati rice

b) Pasta and Yoghurt

c) Polished white rice and sugary foods

d) Soy products and lentils

Skip to question 22

5. Attitudes towards low-GI foods.

I. To what extent do you agree with the statement, "Incorporating low-GI foods into your diet is important for maintaining a good, healthy life."

Mark only one oval.

a) Strongly agree

b) Agree

c) Neutral

d) Disagree

e) Strongly disagree

II. How confident are you in your ability to identify low-GI foods?

Mark only one oval.

a) Very confident

b) Confident

c) Neutral

d) Not confident

e) Not confident at all

III. Do you agree that consuming low-GI foods can help in weight loss, preventing chronic diseases like diabetes, heart disease and help in improving overall health and well-being?

Mark only one oval.

a) Strongly agree

b) Agree

c) Neutral

d) Disagree

e) Strongly disagree

IV. How important is it for you to control your blood sugar levels?

Mark only one oval.

a) Very important

b) Somewhat important

c) Neutral

d) Normally not important

e) Not important at all

V. Would you be interested in learning more about the health benefits of low-GI foods?

Mark only one oval.

a) Very interested

b) Somewhat interested

c) Neutral

d) Not interested

e) Not interested at all

VI. Would you be willing to make dietary changes to incorporate more low-GI foods into your daily meals?

Mark only one oval.

a) Definitely, I already prioritize low-GI foods in my diet

b) I'm open to trying low-GI foods

c) Maybe, if it's convenient

d) No, I prefer my current diet

e) I haven’t considered it /Not at all

VII. Do you think that low-GI foods are more expensive than high-GI foods?

Mark only one oval.

a) Yes, definitely

b) Yes, but it's worth the cost

c) No, prices are similar

d) No, they're cheaper

e) No, they're very cheap

VIII. How do you perceive the convenience of preparing meals with low-GI foods?

Mark only one oval.

a) Very convenient

b) Somewhat convenient

c) Neutral

d) Inconvenient

e) Very inconvenient

IX. How do you perceive the taste of low-GI foods compared to high-GI foods?

Mark only one oval.

a) More flavorful

b) Flavorful

c) About the same

d) Less flavorful

e) Very little flavour

X. Why do you like to consume low-GI foods?

Please scroll to the right and tick an answer for each row.

Mark only one per row.

Strongly agree

Agree

Neutral

Disagree

Strongly disagree

• To reduce the risk of non-communicable diseases, especially diabetes and heart disease.

• To avoid present health issues.

• High nutritional values and health benefits.

• To maintain better health.

• To control blood sugar levels, blood pressure and cholesterol levels

• For weight management.

• Diabetes Management.

• To reduce the risk of non-communicable diseases, especially diabetes and heart disease.

• To avoid present health issues.

• High nutritional values and health benefits.

• To maintain better health.

• To control blood sugar levels, blood pressure and cholesterol levels.

• For weight management.

• Diabetes Management

6. Practices regarding low-GI foods:

I. How often do you consume foods that you know are low GI?

Mark only one oval.

a) Rarely or never

b) Once a week

c) Several times per week

d) Daily

II. Have you ever used YouTube, Facebook, WhatsApp or the internet to search for information about low-GI foods?

Mark only one oval.

a) Never

b) Rarely

c) Sometimes

d) Always

III. How often do you consume foods like whole grains, legumes, fruits and vegetables?

Mark only one oval.

a) Rarely or never

b) Once a week

c) Several times a week

d) Daily

IV. How often do you check the GI of foods before consuming them?

Mark only one oval.

a) Never

b) Occationally

c) Sometimes

d) Always

V. Do you plan your daily meals to include a balance of low GI foods?

Mark only one oval.

a) Never

b) Occasionally

c) Sometimes/whenever possible

d) Always / Daily

VI. Do you normally read food labels to check for the glycemic index of products?

Mark only one oval.

a) Never

b) Rarely

c) Sometimes

d) Always

VII. How often do you consume processed foods and sugary snacks & beverages?

Mark only one oval.

a) Never

b) Occasionally

c) Several times per week

d) Daily

VIII. Do you usually eat out at restaurants for your main meals?

Mark only one oval.

a) Never

b) Occasionally

c) Several times per week

d) Daily

IX. Do you prefer to snack on low-GI foods like nuts and seeds?

Mark only one oval.

a) Never

b) somewhat interested

c) Interested

d) Very interested

X. Do you find it easy to incorporate low-GI foods like whole grains and legumes into your meals?

Mark only one oval.

a) It is very challenging

b) It can be challenging, but I'm trying

c) It's relatively easy

d) It's very easy

XI. How often do you consume processed meat/ products and high-fat dairy products?

Mark only one oval.

a) Rarely or never

b) Occasionally

c) Several times a week

d) Daily

XII. Do you usually snack on fruits or vegetables?

Mark only one oval.

a) Rarely or never

b) Occasionally

c) Several times a week

d) Daily

7. Would you like to participate in our post-evaluation questionnaire after our intervention programme on low-GI foods?

Mark only one oval.

Yes

No

If yes, please mention your Email address ___________________________________

and Phone number (Whatsapp) _____________________.

Nobody sees your details. We would like to send the details of the intervention programmes and the post-evaluation questionnaire. Thank you for taking the time to participate in this survey. Your responses are invaluable to us and will contribute significantly to understanding the factors influencing the consumption of low-GI foods.

## References

[1] Ediriweera DS, Karunapema P, Pathmeswaran A, Arnold M (2018). Increase in premature mortality due to non-communicable diseases in Sri Lanka during the first decade of the twenty-first century. BMC Public Health.

[2] Rannan-Eliya RP, Wijemunige N, Perera P (2023). Prevalence of diabetes and pre-diabetes in Sri Lanka: a new global hotspot-estimates from the Sri Lanka Health and Ageing Survey 2018/2019. BMJ Open Diabetes Res Care.

[3] Jayatissa R, Jayawardana R, Abeysinghe D, De Silva KH (2025). Diet healthiness and double burden of malnutrition among women aged 15-49 years: a global monitoring tool approach using national dietary data in Sri Lanka. BMJ Nutr Prev Health.

[4] Medagama A, Widanapathirana H (2015). An appraisal of food serving characteristics among patients with type 2 diabetes mellitus attending a tertiary care diabetes facility in Sri Lanka. Int Arch Med.

[5] Senadheera SP, Ekanayake S, Wanigatunge C (2016). Dietary habits of type 2 diabetes patients: variety and frequency of food intake. J Nutr Metab.

[6] Yari Z, Behrouz V, Zand H, Pourvali K (2020). New insight into diabetes management: from glycemic index to dietary insulin index. Curr Diabetes Rev.

[7] Rajabi S, Mazloom Z, Zamani A, Tabatabaee HR (2015). Effect of low glycemic index diet versus metformin on metabolic syndrome. Int J Endocrinol Metab.

[8] Clar C, Al-Khudairy L, Loveman E (2017). Low glycaemic index diets for the prevention of cardiovascular disease. Cochrane Database Syst Rev.

[9] Ni C, Jia Q, Ding G, Wu X, Yang M (2022). Low-glycemic index diets as an intervention in metabolic diseases: a systematic review and meta-analysis. Nutrients.

[10] Somaratne GM, Prasantha BD, Dunuwila GR, Chandrasekara A, Wijesinghe DG, Gunasekara DC (2017). Effect of polishing on glycemic index and antioxidant properties of red and white basmati rice. Food Chem.

[11] Arachchilage DL, Ekanayake S (2024). Effect of cooking and in vivo glycemic response of Sri Lankan traditional rice: a source of sustainable and underutilized functional food. Curr Res Nutr Food Sci.

[12] Hettiaratchi UP, Ekanayake S, Welihinda J (2011). Sri Lankan rice mixed meals: effect on glycaemic index and contribution to daily dietary fibre requirement. Malays J Nutr.

[13] Avedzi HM, Mathe N, Storey K, Johnson JA, Johnson ST (2018). Examining sex differences in glycemic index knowledge and intake among individuals with type 2 diabetes. Prim Care Diabetes.

[14] Asmelash D, Abdu N, Tefera S, Baynes HW, Derbew C (2019). Knowledge, attitude, and practice towards glycemic control and its associated factors among diabetes mellitus patients. J Diabetes Res.

[15] Salwathura A, Ahmed F (2023). Dietary pattern, nutrition-related knowledge and attitudes of working women in Western Province, Sri Lanka. Nutrients.

[16] Weerasekara PC, Withanachchi CR, Ginigaddara GA, Ploeger A (2020). Food and nutrition-related knowledge, attitudes, and practices among reproductive-age women in marginalized areas in Sri Lanka. Int J Environ Res Public Health.

[17] Waidyatilaka I, de Silva A, de Lanerolle-Dias M, Wickremasinghe R, Atukorala S, Somasundaram N, Lanerolle P (2014). Lifestyle patterns and dysglycaemic risk in urban Sri Lankan women. Br J Nutr.

[18] Amarasekara P, de Silva A, Swarnamali H, Senarath U, Katulanda P (2016). Knowledge, attitudes, and practices on lifestyle and cardiovascular risk factors among metabolic syndrome patients in an urban tertiary care institute in Sri Lanka. Asia Pac J Public Health.

[19] Chiavaroli L, Lee D, Ahmed A (2021). Effect of low glycaemic index or load dietary patterns on glycaemic control and cardiometabolic risk factors in diabetes: systematic review and meta-analysis of randomised controlled trials. BMJ.

[20] Koyratty N, Nwabuikwi O, Silva R, Hess SY, Olney DK (2026). Diets, fruit and vegetable intake, and nutritional status in Sri Lanka: a scoping review. Matern Child Nutr.

[21] Koch F, Hoffmann I, Claupein E (2021). Types of nutrition knowledge, their socio-demographic determinants and their association with food consumption: results of the NEMONIt study. Front Nutr.

[22] Cortes ML, Louzado JA, Oliveira MG (2021). Unhealthy food and psychological stress: the association between ultra-processed food consumption and perceived stress in working-class young adults. Int J Environ Res Public Health.

[23] Whatnall M, Clarke E, Collins CE, Pursey K, Burrows T (2022). Ultra-processed food intakes associated with 'food addiction' in young adults. Appetite.

[24] Schmidt SK, Hemmestad L, MacDonald CS, Langberg H, Valentiner LS (2020). Motivation and barriers to maintaining lifestyle changes in patients with type 2 diabetes after an intensive lifestyle intervention (the U-TURN trial): a longitudinal qualitative study. Int J Environ Res Public Health.

[25] Grant SM, Wolever TM (2011). Perceived barriers to application of glycaemic index: valid concerns or lost in translation?. Nutrients.

[26] Jhaveri NR, Poveda NE, Kachwaha S, Comeau DL, Nguyen PH, Young MF (2023). Opportunities and barriers for maternal nutrition behavior change: an in-depth qualitative analysis of pregnant women and their families in Uttar Pradesh, India. Front Nutr.

[27] Perera T, Subasinghe GT, Pathirana T (2022). Consumer knowledge, perceptions, attitudes and practices on the use of nutrition labeling including traffic light labeling (TLL) system in Sri Lanka. Curr Dev Nutr.

